# The impact of natural disaster on mental health and how to deal with it?

**DOI:** 10.1192/j.eurpsy.2024.126

**Published:** 2024-08-27

**Authors:** M. Rojnic Kuzman

**Affiliations:** Zagreb University Hospital Centre and the Zagreb School of Medicine, Zagreb, Croatia

## Abstract

**Abstract:**

Natural disasters are and will continue to represent a great challenge in addressing mental health issues globally. The most devastating recent (earthquakes on 6th February 2023 in Turkey and Syria) caused death of more than 55,000 people, injury of about 100,000 people and loss of property, overall affecting millions of people. Moreover, in the last several years in Europe, they came in a form of double disasters (for example coupled with the COVID-19 pandemic) and pointed out the unpreparedness of the health (including mental health) sectors for the emergency situations.

However, in going through these experiences, we also learnt some of the practices that proved effective – including the fast creation of collaborative networks on a larger scale that also allowed fast spread of good practices and practical organisation of help. As a practical example of it - verbalized by the mental health professionals from Turkey through the Council of National Psychiatric Associations of the European Psychiatric Association, we organized a webinar delivered by experienced clincians, trauma experts and experts with lived experince in the earthquake zones. However, structural -implementation of mental health policies that focus on prevention and improving crisis response in care delivery are important to support populations affected by natural disasters to prevent the trauma sequel.

**Disclosure of Interest:**

None Declared

